# Echocardiographic Study of Ventricular Septal Defect in 1- to 12-Year-Old Children Visiting a Tertiary Care Center in Patna, India

**DOI:** 10.7759/cureus.46363

**Published:** 2023-10-02

**Authors:** Amish Kumar, Pankaj Kumar

**Affiliations:** 1 Department of Anatomy, Indira Gandhi Institute of Medical Sciences, Patna, IND; 2 Department of Anatomy, Narayan Medical College and Hospital, Patna, IND

**Keywords:** arterioventricular (av) canal, chronic heart disease (chd), ventricular septal defect (vsd), pulmonary arterial hypertension, muscular septum, infective endocarditis

## Abstract

Background

Symptoms of serious heart problems present at birth often appear during the first few days, weeks, or months of a child's life. Shunt formation between the left and right ventricle is a crucial component of the pathophysiology of ventral septal defects.

Objectives

We aim to determine the most frequent types of ventricular septal defect (VSD) by echocardiography and whether there is any gender variation in the type of ventricular septal defect.

Material and methods

A total of 100 children who were clinically suspected of or diagnosed with VSD at the age of 1-12 years were enrolled in this study. The septum of the atrioventricular (AV) canal, the muscular septum, and the parietal band of the distal conal septum were evaluated by color Doppler. Ventricular septal defect (VSD) size and kind are similarly impacted by the 2D echo mode. The size and site of the VSD, associated congenital anomaly, and significant morphological changes in ventricular cavities, gender discrimination, and relation-specific types of ventricular septal defect were observed.

Results

A total of 100 VSD children presented with clinical symptoms of fast breathing, retraction of the chest, cough, cyanosis, fever, difficulty during feeding, cyanotic spell, chest pain, and edema at 65%, 62%, 54%, 52%, 54%, 29%, 9%, 11%, and 4%, respectively.

Conclusion

Early diagnosis is essential for effective medical care of diseases such as infective endocarditis (IE), which is present in some cases of VSD, and the avoidance of persistent pulmonary veno-occlusive disease (PVOD).

## Introduction

Congenital heart defects, which are significant cardiac conditions present at birth, may manifest their symptoms in the first few days, weeks, or months of a child's life. Biventricular and biatrial heart evolution has been comprehensively studied previously [1]. Congenital heart disease (CHD) is a leading cause of mortality and morbidity among pediatric subjects globally, including India, where approximately 10% of deaths in Indian infants are attributed to CHD. Previous literature data on a global scale reported the incidence of CHD to be in the range of 1-17.5 per 1,000 live births with the most commonly considered evidence being 12 per 10,000 live births [2].

In the Indian scenario, the incidence is four per 1,000 live births, and the prevalence of CHD in India is found to be 2-5 per 1,000 children. Among all reported CHDs, ventricular septal defects (VSDs) alone cause 20%-25% of all reported CHDs and are taken as the most common form of CHD [3].

VSDs are usually divided into four main types depending on their location on the ventricular septum: muscular defect located on the apical and muscular areas of the ventricular septum, atrioventricular (AV) septal defect located on the posterior septum, supracristal defect located on the conal septum in the sub-pulmonary region, and perimembranous defect located in the subaortic region on the membranous ventricular septum [4,5].

Spontaneous closure is seen in many cases of VSD; however, larger septal defects lead to various harmful complications including increased risk for arrhythmia, ventricular dysfunctions, pulmonary arterial hypertension, and/or infective endocarditis (IE) [6].

The creation of a shunt between the left and right ventricles plays a vital part in the pathophysiology of VSD. The direction of the blood flow and the amount of the blood flow through the shunt govern the hemodynamic equation in the ventricular septal defects, which further depict the pulmonary vascular resistance (PVR) and the location and size of the ventricular septal defects [7].

It has become increasingly challenging for clinicians to handle cases of congestive heart disease with an increased attempt to handle cases of CHDs owing to the reported high rates of success in treating CHDs in developed countries [8].

To bring the needed changes in the policies of healthcare, it is vital to comprehensively study the type and extent of CHDs, which largely govern the rate of infant mortality. With the introduction of echocardiography in the field of cardiology, the diagnosis of CHD has been largely improved and has become better. Following its validation, Doppler echocardiography has shown to be a sensitive tool for the diagnosis of ventricular septal defects, which also accurately reflects the hemodynamics of the heart.

The present study aimed to assess the echocardiography results of subjects suspected of VSDs [9,10]. The present study also aimed at assessing the most common type of VSD evaluated by echocardiography, assessing the variation of gender based on the type of VSD, and assessing the VSD frequency with other cardiac congenital anomalies in the given population.

## Materials and methods

The present clinical study was aimed to assess the echocardiography results of subjects suspected of VSDs. The study also aimed at assessing the most common type of VSD evaluated by echocardiography, assessing the variation of gender based on the type of VSD, and assessing the VSD frequency with other cardiac congenital anomalies in the given population, i.e., 100 children who visited the outpatient department of the institution. Ethical clearance was taken from the institutional ethical committee before the initiation of the study (ethical number: 94/IEC/IGIMS/2021).

This study was based on hospital records. This is a retrospective observational research conducted on 100 children who visited the outpatient department of cardiology of Indira Gandhi Institute of Medical Sciences, Patna, Bihar, India. A total of 100 children were enrolled in this study. The study population belonged to the age group of 1-12 years. The study period was from June 2021 to December 2022. Consent was taken from the parents of the subjects.

We have excluded uncomplicated/isolated bicuspid aortic valves to avoid overestimation of CHD prevalence and patients with acquired heart diseases, such as rheumatic heart disease. Ventricular septal defect (VSD) was detected in 100 children who were already receiving treatment for it by utilizing a pediatric echo probe and paying close attention to the three major areas of the interventricular septum (IVS). The septum of the AV canal, the muscular septum, and the parietal band of the distal conal septum were evaluated. To be quickly scanned in 2D mode and color Doppler, ventricular septal defect size and kind are similarly impacted by the 2D echo mode. The blood flow status (ejection fraction, diastolic dysfunction, and left ventricular (LV) systolic dysfunction) may be determined using M mode. We can determine the location of the shunt and whether there are any issues such as pulmonary artery hypertension (PAH) via color Doppler.

The parameters size and site of the VSD, associated congenital anomaly, and significant morphological changes in ventricular cavities, gender discrimination, and relation-specific types of VSD were observed.

Each of the participants was subjected to echocardiography to determine the size of the VSD and the direction of the shunt. An echo machine with a 2D mode facility was utilized to determine the three components of the interventricular septum (IVS) responsible for the various sizes of the defect and the direction of the shunt. These were ascertained with the help of color Doppler.

Statistical analysis

Data were compiled in a Microsoft Excel sheet (Microsoft Corp., Redmond, WA) and described by estimated proportion. Data were presented as tables. Fisher's exact test was used to draw statistical inferences. The Statistical Package for the Social Sciences (SPSS) version 21 (IBM SPSS Statistics, Armonk, NY) was used for data analysis. A p-value of <0.05 was considered statistically significant.

## Results

Regarding age distribution among the study population, of the 100 children diagnosed with VSD, 64% belonged to the 1-5 years age group, 28% belonged to the >5-10 years age group, and 8% belonged to the >10-12 years age group. There were more male children than female children at 60% and 40%, respectively (Table 1).

**Table 1:** Age and gender distribution of study subjects

Parameter	Subgroup	Number of cases	Percentage (%)
Age in years	1-5	64	64
	>5-10	28	28
	>10-12	8	8
Sex	Male	60	60
	Female	40	40
Male-to-female ratio	1.5:1		

A total of 100 VSD children presented with clinical symptoms of fast breathing (65%), retraction of the chest (62%), cough (54%), cyanosis (52%), fever (54%), difficulty during feeding (29%), cyanotic spell (9%), chest pain (11%), and edema (4%) (Table 2).

**Table 2:** Clinical presentation

Clinical presentation	Number of cases	Percentage (%)
Fast breathing	65	65
Retraction of chest	62	62
Cough	54	54
Cyanosis	52	52
Fever	54	54
Difficulty during feeding	29	29
Cyanotic spell	9	9
Chest pain	11	11
Edema	4	4
Total	100	100

The majority of children had a VSD size of <5 mm in diameter (51%), followed by children with a VSD size of ≥5-10 mm in diameter (43%), and children with large VSD size, i.e., >10 mm in diameter (6%) (Table 3).

**Table 3:** Size of VSD VSD: ventricular septal defect

Size of VSD	Number of cases	Percentage (%)
Small (<5 mm)	51	51
Moderate (≥5-10 mm)	43	43
Large (≥10 mm)	6	6
Total	100	100

The types of VSD the children have are presented in Table 4. We found perimembranous VSDs to be the most common congenital heart defect (63%), followed by inlet (1%), outlet (2%), muscular (19%), subaortic (13%), and subpulmonic VSDs (2%) (Table 4).

**Table 4:** Type of VSD VSD: ventricular septal defect

Type of VSD	Number of cases	Percentage (%)
Perimembranous	63	63
Inlet	1	1
Outlet	2	2
Muscular	19	19
Subaortic	13	13
Subpulmonic	2	2
Total	100	100

Out of 60 male cases, perimembranous VSD was found at 61.7%, outlet VSD at 3.3%, muscular and subaortic VSDs at 16.7% each, and subpulmonic VSD at 1.7%. On the other hand, of 40 female cases, 26% had perimembranous VSDs, 2.5% had inlet and subpulmonic VSDs each, 22.5% had muscular VSDs, and 7.5% had subaortic VSDs. There was no significant difference between male and female children and type of VSDs (p=0.423) (Table 5).

**Table 5:** Gender variation in the type of VSD VSD: ventricular septal defect Chi-square value: 4.9401, p-value: 0.423

Type of VSD	Male (n=60)		Female (n=40)	
	Number of cases	Percentage (%)	Number of cases	Percentage (%)
Perimembranous	37	61.7	26	65
Inlet	0	0	1	2.5
Outlet	2	3.3	0	0
Muscular	10	16.7	9	22.5
Subaortic	10	16.7	3	7.5
Subpulmonic	1	1.7	1	2.5
Total	60	100	40	100

## Discussion

The present study assessed pediatric subjects with VSD within the age range of one year to 12 years as VSDs usually close spontaneously by the age of 12 years. The results of the present study reported that on Doppler findings, perimembranous type was the most common VSD type seen in 63% of the subjects, with 93% having L-R shunt, and was smaller in size. Bidirectional blood flow was seen in the majority of subjects with larger shunts. Of all reported complications, nearly 50% were seen in VSD of a smaller type. Complications were sustained by a higher proportion of larger VSDs compared to smaller VSDs. VSD can lead to L-R shunt that can further lead to congestive cardiac failure (CCF) in younger subjects with larger VSD, but in those with smaller VSD, it can be detected accidentally and can remain silent for a considerable amount of time or if they do not close on their own as suggested by Yasmeen et al. in 2007 [11] and Wanni et al. in 2014 [12].

In the present study, the male-to-female ratio was found to be 1.5:1, which was consistent with the studies of Dakkak and Oliver in 2020 [13] and Singh et al. in 2018 [14]. However, the age range was not described by Dakkak and Oliver [13] and Singh et al. [14] who assessed subjects aged 0-18 years. In cases of VSD, male predominance was reported in the studies of Fyler et al. in 1958 [15] and Frescura et al. in 2016 [16]. This difference can be attributed to different selected age ranges for study samples. Also, similar roles can be played by the consent of the female children's parents, gender bias in various studies, and differences in sampling methods.

Rao and Harris [17] suggested the most common type of VSD to be membranous type seen in 80% of subjects, whereas muscular, AV septal, and supracristal types were seen in 5%-20%, 8%, and 5%-7% of cases, respectively. The majority of VSDs seen are single, whereas multiple defects can be seen, depicting a muscular variety known as the Swiss cheese type. A higher prevalence of perimembranous type was also seen in the study by Manuel et al. in 2014 [18].

Out of 60 male cases, perimembranous type VSD was found at 61.7%, outlet VSD at 3.3%, muscular and subaortic VSD at 16.7% each, and subpulmonic VSD at 1.7%. On the other hand, of 40 female cases, 26% had perimembranous VSD, 2.5% had inlet and subpulmonic VSD each, 22.5% had muscular VSD, and 7.5% had subaortic VSD. There was no significant difference between male and female children and type of VSDs. The p-value was 0.423. These studies might have conflicting results attributed to different age ranges, sampling methods, and different size errors. The study by Khokhar et al. [19] suggested that no subject had infective endocarditis. However, 48% of subjects were reported with pulmonary arterial hypertension.

The prevention of pulmonary veno-occlusive disease (PVOD) by adopting any possible measure is vital and important. Surgical closure of VSD of moderate size is usually indicated by LV and left atrium (LA) that are markedly engorged and failure to thrive. Surgical closure is also indicated by a pulmonary-to-systemic flow ratio (Op:Os) of more than 2:1. In the present study, among six VSD defects of large size, 83.4% (n=5) of children reported more complications. It was also seen that mainly three types of complications were seen in the present study, in 20% of subjects, including isolated valvular disease, infective endocarditis, and pulmonary arterial hypertension. The difference was statistically significant at p=0.05.

Limitations

The study population was restricted to a particular geographic region. The number of study participants was less to conclude all types of observations. The dataset was not uniform for each type of variation.

## Conclusions

The present study concludes that most of the ventricular septal defects usually showed a small variety of favorable L-R shunts in the given study population. However, the majority (63%) of the VSDs were of a perimembranous type, which was not prone to spontaneous closure. Therefore, early diagnosis is essential for effective medical care of diseases such as IE, which is present in some cases of VSD, and to avoid persistent PVOD.

As VSD is a fatal condition, without taking heart failure and weight gain management into consideration, the closure of the VSD should be finished before 6-12 months and at most by 18 months.
